# Decolonizing Botany: Indonesia, UNESCO, and the Making of a Global Science

**DOI:** 10.1007/s10739-023-09734-8

**Published:** 2023-10-11

**Authors:** Andrew Goss

**Affiliations:** Department of History, Anthropology, and Philosophy, Augusta University, Augusta, GA USA

**Keywords:** Botany, Indonesia, Decolonization, Internationalism, Scientific cooperation, UNESCO

## Abstract

Decolonization created new opportunities for international scientific research collaboration. In Indonesia this began in the late 1940s, as Indonesian scientists and officials sought to remake the formerly colonial botanical gardens in the city of Bogor into an international research center. Indonesia sponsored the Flora Malesiana project, a flora of all of island Southeast Asia. This project was formally centered in Bogor, Indonesia, with participation from tropical botanists from around the world. The international orientation of Indonesian science led to the establishment of one of UNESCO’s Field Science Co-operation Offices in Jakarta, and to a period of close collaboration between Indonesian botanists and UNESCO. This paper examines the importance of UNESCO’s Humid Tropics research program, which initially provided further opportunities for Indonesian botanists to participate in international scientific networks. The paper concludes by showing that the Humid Tropics program led to the slow erosion of Indonesian agency and authority over tropical botany, and the assertion of Western control and management over tropical botany research.

## Introduction: Decolonization and Global Science

In the decades after 1945 scientists from across Asia, Latin America, and Africa engaged in sustained efforts to globalize science. Decolonization created numerous new possibilities for collaboration and exchange, which had been difficult to create and sustain across imperial boundaries. Scientists in independent and sovereign nations outside of Europe and North America strove to become part of a global community of scientists. They engaged with the world of science, no longer as collectors, assistants, or research subjects, but now as researchers, subject experts, and scientists. In this paper, I show how scientific globalists began to form cooperative collaborations with scientists in other countries, joining international scientific organizations, and seeking to present, publish, and publicize their research to a global audience. These scientists envisioned and built new networks, centered not on the Western metropoles but on their own scientific institutions, linked together for the purpose of generating knowledge meaningful to a global humanity. In this way, decolonization led to both the creation of national cultures of science *and* new global scientific networks of collaboration and cooperation (Sivasundaram 2010).

At the same time, in a parallel process, scientists in Europe and North America began using the institutions of the United Nations (UN) to create new forms of international cooperation in science. They were motivated not so much by decolonization as by the belief that after years of world conflict science properly implemented and applied, would build a culture benefitting people worldwide. The evolutionary biologist Julian Huxley became the first Director-General of United Nations Educational, Scientific and Cultural Organization (UNESCO) in 1946 (Waters and Van Helden 1992; Smocovitis 2016). He saw scientific internationalism not only as an antidote to the jingoism that had perverted science in Germany, but as a way for creating global welfare on the basis of science. He and his team set out to create new frameworks for coordinating scientific education and research across national borders, for the benefit of human culture generally (Huxley 1946). Although led by Western scientists, this scientific internationalism was serious about including scientists from outside Europe and North America, and building scientific institutions in South America, Africa, and Asia. For a brief period in the late 1940s and early 1950s, scientists from Asia, Africa, and South America worked together with the Western scientific internationalists to build a new system of global science, one that seemed to hold the promise of transcending the imperialism, nationalism, and chauvinism of the years before 1945.

In this paper, I explore this short-lived period of idealism in the world of science and detail the path to its demise. I will do so in an Indonesian context, which makes for an excellent place to study this in detail. Indonesia gained independence in 1949 after one of the first anti-colonial and nationalist revolts in Asia. Moreover, Indonesia in the 1950s was a center of experimentation in new global collaborations. Most famously this led to Indonesia hosting the Bandung Conference of Asian and African states in 1955, which sought to create new economic and cultural networks across decolonized Asia and Africa.^1^ Independent Indonesia was also deliberate in participating in global networks of science, especially in botany. Botany—alongside agricultural science and forestry—had the best-established institutions, centered on the Botanical Gardens in Bogor (previously Buitenzorg), known after 1948 as the Kebun Raya Indonesia. Throughout the 1950s Indonesian botanists decolonized the management, resources, collections, and research agendas of the former colonial institutions in and around Bogor. They also endeavored to place Indonesia at the center of a global network of tropical botany. Indonesian botanists started new collaborative research projects, traveled to international conferences, and sent their students abroad for graduate studies. The Kebun Raya Indonesia funded a new international research project, the Flora Malesiana, a comprehensive flora of all of tropical Southeast Asia, reaching beyond Indonesian borders in both its scope and its research collaborators. Botanists in Bogor resumed collecting trips and the processing of the herbarium collections, which had largely stopped during the 1940s. And Indonesian botanists engaged with the scientific opportunities created by UN agencies, especially UNESCO, which established a science office in the capital of Jakarta in 1951. In the 1950s, Indonesian scientists felt poised to change the face of tropical biology, breaking its colonial focus on serving imperial goals, and now generating knowledge meaningful to Indonesia, tropical Asia, and the rest of the world.

Decolonization in the 1940s and 1950s thus created political opportunities for designing new national science policies in Asia. Politically this was driven by an effort to leverage science for technologically driven development (Phalkey 2013; Phalkey and Wang 2016; Moon 1998). The involved scientists intended for these policies to generate national scientific achievements with international visibility. For example, after 1947 Indian physicists believed creating internationally credible experiments was critical to developing self-reliance in science and technology (Abraham 1996). Jawaharlal Nehru, India’s first Prime Minister and himself trained in the sciences as an undergraduate at University of Cambridge sought to place science at the center of a modern Indian nation, with the intention that Indian science would improve the welfare of India and Indians (Arnold 2013). In the Philippines, natural history collections created opportunities to imagine a national identity built out of a global view of the archipelago’s nature (Pagunsan 2020). Even as scientists, politicians, and policy-makers in the post-colonial nations of Asia created national scientific cultures, they continued to strive to integrate their national science with global networks and traditions (Prakash 1999). Decolonization did not lead to self-isolation in science, but instead was an opportunity to develop independent traditions of science while also participating in a new global science.

How did these post-colonial scientists form international networks of science? A promising line of interpretation suggests global science after 1945 originated in the interwar scientific internationalism of European left-leaning scientists, who had promoted the universality of science. New scientific organizations, such as the International Council of Scientific Unions, founded in 1931, promoted scientific cooperation as well as a socialist view that science was a means for solving the problems of humanity as a whole. Scientific internationalists saw nationalism, jingoism, and imperialism, epitomized by the rise of Nazism in Germany, as the enemy of scientific advancement (Somsen 2008). After the defeat of the Nazis, new UN agencies became vehicles for testing scientific internationalism at a larger scale. In its early years, science was a prominent component of UNESCO’s mission, especially from 1946 to 1948, when Huxley was its Director-General. Huxley’s idealistic belief in the power of knowledge to improve the world, as well as his continued conviction that future human culture and politics could be managed under the care of scientists such as himself, gave him confidence that UNESCO could create “One World” through scientific internationalism (Sluga 2010). Huxley chose Joseph Needham as the first head of UNESCO’s natural sciences department. Needham was a research chemist who in the late 1930s had grown interested in the history of Chinese science and technology while working with Chinese graduate students at University of Cambridge. During World War II, Needham had managed the Sino-British Scientific Cooperation Office in China, and this experience had convinced him that scientific internationalism had so far failed to account for the contributions of Chinese and other traditions of science (Mougey 2017). In 1945 Needham had been the leading advocate for adding science to UNESCO’s core mission, as planning during the war had intended for it to cover education and culture without an explicit reference to science (Andersen 2021; Archibald 2006). After 1946 Needham had the backing of Huxley and UNESCO as he sought to build a global system of science which cut across imperial and national divides, and included colonial and post-colonial scientists who had little scientific infrastructure (Petitjean 2006a). At UNESCO Needham developed his “Periphery Principle,” which sought to build research institutions in Latin America and newly decolonized nations of Asia (Petitjean 2006c). An early effort of Needham’s was to create international scientific laboratories, which resulted in efforts during the late 1940s to create the International Institute for the Hylean Amazon in Brazil (Mougey 2018; Petitjean et al. 2006). The Field Science Co-Operation Offices, discussed below, flowed out of these early experiences of creating new forms of international science centered on UNESCO.

Despite the efforts by UN-affiliated agencies to invent new forms of intellectual cooperation after 1945, they did so by creating institutions that replicated elements of colonial and imperial culture (Mazowar 2009). Joseph Hodge has shown that in the 1950s and 1960s, organizations such as the Food and Agricultural Organization of the UN (FAO) and the World Food Organization (WFO), hired large numbers of former European colonial scientists to lead technical assistance and other international developmental projects (Hodge 2010). At UNESCO, Needham, notwithstanding his leftist politics, envisioned a quasi-imperial scientific network, centralized in Europe under UNESCO’s scientific management and reaching out to the rest of the world, ultimately leading to what Thomas Mougey identifies as technocratic and neocolonial outcomes (Mougey 2021). Casper Andersen, Perrin Selcer and others have argued that UNESCO was a crucial force in decolonizing science, but did so by creating an international community of scientists whose knowledge was descended from colonial models (Andersen 2021; Cutroni 2016; Selcer 2015). Geert Somsen, extending an argument made by Lorraine Daston, demonstrates that beginning in the Cold War, historians of science have viewed science as a uniquely European initiative, a gift they offered to the rest of the world (Somsen 2008; Daston 2006). Despite their global outlook, Western scientific internationalists after the war never dropped a belief in European exceptionalism in science and technology. Emblematic of this approach is George Basalla’s well-known 1967 article which, although usually remembered for its arguments about colonial science and the diffusion theory, was a prescriptive set of guidelines for building independent and national scientific traditions along European models. Basalla argued that to be successful in science, newly independent countries would need to adopt European attributes, like separating science and religion, eradicating superstitions, and providing adequate government funding (Basalla 1967). Pratik Chakrabarti, in a recent review situating empire and science, reminds us that the continued Eurocentrism of history of science, despite sustained efforts to decolonize the field, is itself evidence that science is an imperial epistemology (Chakrabarti 2021).

The focus of much of the historiography on the decolonization of science has investigated how scientists in Europe and North America sought to invent new international systems of scientific cooperation. Despite a voluminous literature on the close ties between science and empire (Goss 2021; Hodge 2011; MacLeod 2000; Palladino and Worboys 1993), there are far fewer in-depth studies of how imperial science decolonized in the former colonies. Those studies that do exist have stressed continuities from imperial to post-colonial science. For example, Raf de Bont points to the continued influence of the older rhetoric of scientific internationalism in independent Congo, where after independence in 1960, the transnational network supporting the Albert National Park survived, with the help of UNESCO and international conservation NGOs (De Bont 2017). In the early 1960s, Julian Huxley, as part of UNESCO’s new conservation initiatives, proposed a system of African national parks, run by the newly independent African governments but supported by the global conservationists. New research has shown that Huxley’s effort to preserve African nature for the benefit of global humanity was a continuation and elaboration of colonial era practices of ecological fieldwork in British Africa (Sommer 2016; Tilley 2011). And other studies show that within conservation biology in Africa during the era of decolonization, Western scientists and conservationists continued to hold dominant positions within ecology (Andersen 2016; De Bont 2020). In Asia, the Pacific Science Congresses, started in 1920, were intended to coordinate scientific research in the Pacific, and during the Cold War, these brought US, Canadian, and Australian scientists together with Asian researchers (Rehbock 1991).

These studies demonstrate continuity between the scientific internationalism of the interwar period and the post-war global science initiatives, and suggest the existence of colonial and imperial undertones in the articulation of post-war scientific universalism, while largely ignoring the story of the recently decolonized themselves. My argument builds on these findings, by investigating how scientists in newly decolonized countries participated as local experts seeking agency in global scientific cooperative efforts. I examine both the continuities from the colonial era, including the continued prominence of colonial scientists in global science, but also the changes resulting from Asian scientists’ participation in international networks of scholarship, and the complex role played by scientific internationalism, in particular those organized under UNESCO.

## Indonesian Science

Answering the broader question of how science decolonized after 1945 means looking not just at the efforts in Paris or London to orchestrate universalism. It requires greater in-depth studies of the decolonization of science, at the sites where global networks of scientific cooperation were being enacted. A study of Indonesian science in the 1950s contributes to this literature by examining the cooperation between colonial, Indonesian, and UNESCO scientists, right when science seemed capable of truly decolonizing and globalizing. Continuing the study into the 1960s shows the struggle for respect and authority in their scientific fields by scientists who had until recently been colonial subjects barred from Eurocentric scientific society. After 1950, Indonesian scientists were enthusiastic globalists, seeking not just new collaborative relationships, but endeavoring to establish Indonesia as a center of science, in particular in tropical botany. In the 1950s, this effort showed promise, as Indonesian biologists started new research projects and presented their findings at international scientific meetings. They also established a productive relationship with UNESCO, and found ways to leverage UNESCO resources to advance Indonesia as a hub of tropical botany. Prior to 1945, Dutch colonial scientists had shown little interest in scientific internationalism, but the end of the Dutch Empire meant either a return to the Netherlands (and uncertain employment as there were few jobs in tropical botany), or participation in new positions in Indonesia, which a number of them pursued. Nonetheless, Indonesia did not emerge as a center of global tropical botany, despite its having both the geographical location in the tropics, and housing important and extensive botanical collections at the herbarium in Bogor. Ultimately control over global botany came to rest with tropical biologists at Western institutions, who gained power over funding and resources in tropical biology, despite working well north of the tropics and having lost their privileged access to tropical nature. I explore the reasons for this, detailing both Indonesia’s slow drift away from global interactions with the West, and the concurrent success of European and North American botanists in shifting UNESCO’s scientific globalization towards privileging Western institutions.

The Indonesian Revolution (1945–1949) led to Dutch colonial scientists and administrators in Bogor being gradually replaced by Indonesians, many of whom had previously worked as subordinates within colonial science.^2^ During the colonial period the Dutch had made a considerable investment in science and scientific research, especially in biology, agriculture, and forestry. After 1903, this had included training research assistants and agricultural extension officers at an Agricultural College attached to the Botanical Gardens (Goss 2011). During the first few decades of the twentieth century, the Botanical Gardens expanded to include numerous specialized institutions dedicated to forestry, export-crop research, and indigenous agriculture as well as scientific research (Van der Schoor 2012; Moon 2007; Maat 2001). Although the scientists and administrators were all, without exception, European, there were numerous Indonesians who worked as research assistants, some of whom had been trained at the Agricultural College. A very small number, less than ten, had received academic training in Holland, at the Agricultural School in Wageningen (Messner 1994). In the late 1930s, there were about 400 Indonesian college graduates, but most of them were physicians (Dwidjoseputro 1970).

The Indonesian Revolution also created new opportunities for Indonesian intellectuals and professionals to control their fields. Physicians played an important role during and immediately after the revolution in establishing the political and institutional basis of medicine and science in Indonesia (Pols 2018, 2021). Starting in 1947, the Dutch colonial authorities began, in territory that they controlled, to create a federal governance system in which some power was held by Indonesian officials. In 1948, authority over science came under the Ministry of Agriculture and Fisheries, directed after March of 1948 by Wisaksono Wirjodihardjo. Wirjodihardjo was a graduate of the Agricultural College in Bogor, an Indonesian soil researcher, and a moderate Indonesian nationalist who in 1947 and early 1948 had been mayor of Bogor.^3^ Wirjodihardjo had responsibility over the Bogor Botanical Gardens, and was interested in transforming the Bogor scientific institutes into Indonesian research centers.^4^ Wirjodihardjo was part of a cohort of moderate nationalists, who prior to the war had worked within the Dutch system. In the late 1930s, these Indonesian nationalists and intellectuals had noted the lack of scientific achievement for Indonesia, and argued that they needed to find ways for Indonesians to participate in the international endeavor of science, which would, they expected, lead to the intellectual emancipation of the Indonesian nation (Ratoe Langie 1938). While colonial science in the Netherlands East Indies had been closely integrated into colonial governance, and was a means for generating practical and ideally profitable information and knowledge about colonial nature, Indonesian research science was to be a way for the Indonesian nation to contribute to the larger world of scientific research and thus garner respect for the country’s scientific capacity.

Wirjodihardjo and others after 1948 began to look for ways to establish the global reputation and legitimacy of Indonesian science. Botany was an obvious place to start. At first this meant gaining control over the former colonial institutes. Koesnoto Setyodiwiryo, an agricultural engineer who had been one of the few Indonesians to attend the Wageningen Agricultural School in the Netherlands, became director of the Kebun Raya in early 1950. In addition to reinventing the formerly colonial Botanical Gardens as a national institution (Goss 2018), Koesnoto sought ways to have the Kebun Raya participate in science that transcended national borders. With no academically trained Indonesian biologists, however, and few connections to scientists outside of Indonesia, this meant continued collaboration with formerly Dutch colonial researchers. Dutch colonial scientific managers were unneeded, and D. F. van Slooten, who had preceded Koesnoto as director of the Botanical Gardens, returned to the Netherlands when Koesnoto became director.^5^ But a number of Dutch scientists remained in Bogor at the Kebun Raya, now working as researchers for the Indonesian government. This included M. A. Donk, the head of the herbarium, J. Ruinen, who oversaw the Treub laboratory, and A. J. G. H. Kostermans, in the forestry department in Bogor. Koesnoto’s most important collaborator, and his main conduit to the world of tropical botany, was the botanist Kees van Steenis, who had worked as a botanist in Bogor since 1928. Together they started the Flora Malesiana research project in 1950.

## The Flora Malesiana

The *Flora Malesiana* was, and is, a publication series that strives to name and inventory all the vascular plants of island Southeast Asia. It was always understood to be a massive project, one that would involve extensive and long-term participation from botanists in Indonesia, the Netherlands, tropical Asia, and elsewhere.^6^ It was originally conceived by Van Steenis, who had been talking about it since the mid-1930s.^7^ It was begun in the 1940s, when Van Steenis, with his wife M. J. van Steenis-Kruseman and Bogor herbarium colleague H. de Wit, created the framework for the project. Still, after 1945 it had no formal support from the Dutch scientific leadership in Bogor. It only became a formally funded project with government support after Wisaksono and Koesnoto became the scientific leaders in Jakarta and Bogor. The Indonesian government supported it because it was a credible way for Indonesian political and scientific officials to place Indonesia at the center of an international scientific research project. And although it was directed by a former colonial scientist—Van Steenis—Indonesian scientists saw it as a way of leaving the practices of colonial botany behind.

The Flora Malesiana project, while originally conceived by a colonial scientist, had never found a place within Dutch colonial science. Dutch colonial scientists and officials had repeatedly dismissed Van Steenis’s dream of a complete flora of island Southeast Asia as unrealistically large in scope, not profitable either economically or scientifically, and the wrong direction for Dutch colonial science during the depression. Moreover, there was almost no interest at that time amongst the scientific administrators in international collaboration, a key attribute of Van Steenis’s vision. A generation before Van Steenis, the Bogor Botanical Gardens had become famous in Europe for its visitor’s laboratory, where European and North American scientists could conduct tropical flora research, with the results published under the visiting scientist’s name (Goss 2011; Cittadino 1990). True collaboration in scientific research was exceptional and partial: for example, the lavishly funded Smithsonian Sterling-expedition to New Guinea in 1926 was a joint effort by Dutch and US scientists. But even here, while the ethnographic collecting on that expedition was shared, the research questions were not, and the Dutch and US scientists did not work together to analyze the data or publish the results (Taylor 2006). Moreover, botanists in neighboring colonial territories such as Singapore saw the Dutch colonial Botanical Gardens as a rival to be emulated and bested, not as a place for collaboration (Barnard 2016). And by the 1930s, as a result of budget cuts, protectionist politics, and new scientific leadership, there were few visitors coming to Bogor, and there was no real interest in collaboration amongst the Garden’s leadership. At this time, Van Steenis did interact with a network of botanists organized through Frans Verdoorn’s New York publication *Chronica Botanica*, where he published an essay lamenting the Dutch colonial state’s lack of interest in internationalist science (Van Steenis 1935b).^8^ But Verdoorn and others in the United States had no influence over Dutch colonial science.

Van Steenis’s international flora was also dismissed as wrong-headed by systematists in Holland. Van Steenis was in the 1930s the junior botanist in Bogor, and he had no collaborators on his project—moreover his formal position was as the economic botanist, not as the systematist. In Holland, H. J. Lam, who in the 1930s was the director of the Leiden Rijksherbarium but had started his career in Bogor in 1919, published a critical appraisal of Van Steenis’s work (Lam 1936). In a private letter, Lam upbraided Van Steenis for carelessly dismissing a colleague’s work.^9^ Unbeknownst to Van Steenis, Lam and other Dutch botanists were actively working against him and the Flora Malesiana project in the late 1930s, as they believed it was detrimental to rebuilding the Botanical Gardens as a center of colonial science.^10^ Separately, Van Steenis’s PhD advisor A. A. Pulle in 1938 had proposed that it was high time to update the last colonial flora written in the middle of the nineteenth century, and that it could be done from Utrecht University, but funded by the Dutch colonial budget (Pulle 1938). Although this proposal, with its focus only on the Dutch colony, was more in line with colonial science than Van Steenis’s broader flora, it went nowhere. In the major institutional reorganization of the Botanical Gardens pushed through in early 1940, the focus was coordinating scientific research across the Dutch colony for the purpose of more effective economic development. The formal plans for this newly expanded Botanical Gardens—largely unrealized because of the German occupation of the Netherlands a few months later—only mentioned in passing the need for an updated flora of the Dutch colony, and nothing about a more ambitious survey of Southeast Asian plant life.^11^

It was only the displacement of Dutch colonial science by the Japanese in 1942 that created the first opportunity for Van Steenis to work full-time on the Flora Malesiana, this time under Japanese management. For three years Van Steenis, Van Steenis-Kruseman, and De Wit, worked for Japanese scientists, whose interest in general and summary taxonomic writings about the flora of the Japanese possessions in island Southeast Asia was similar to the idea of The Flora Malesiana.^12^ As a result of this research agenda, Van Steenis and his Dutch collaborators finished many of the essays which would form the first essays published in the *Flora Malesiana* a few years later.^13^ After the Japanese defeat and the return of Dutch power in Bogor, Van Steenis was thwarted by rivals and officials in the Dutch colony, who continued to sideline him and eventually prevented him from receiving an official scientific position in Buitenzorg in 1948.^14^ Van Steenis had shown no sympathies for the Indonesian Republic prior to 1949, but he had by the late 1940s realized that the Flora Malesiana project had no real support from the Dutch colonial authorities.^15^ And so when during the course of 1949 Dutch colonial power in Indonesia fully collapsed, Van Steenis was ready to work with the new administrators and managers, who now were Indonesian nationalists, politicians, and scientists, such as Wisaksono and Koesnoto.^16^

One thing that appealed to Wisaksono, Koesnoto and other Indonesian scientists was the international network that Van Steenis had been building around the Flora Malesiana project. Between 1946 and 1949 Van Steenis had worked to drum -up international support for the project, writing thousands of letters to botanical institutes around the world.^17^ During the winter of 1946–1947 Van Steenis was in the United States checking libraries and collections, spreading the word of the Flora Malesiana, and seeking collaborators as well as funders. Botanists at Harvard, including Elmer Merrill, were enthusiastic and agreed to cooperate, although at the New York Botanical Garden they apparently thought Van Steenis a fantasist for starting such an ambitious project (Van Steenis-Kruseman 1988). Although he received no promise of monetary support in the US or elsewhere, he raised awareness and interest, and had built the frame of an international network of potential collaborators and supporters amongst specialists in tropical botany. In July 1947 he mailed out 150 copies of the first issue of the English language *Flora Malesiana Bulletin*, a newsletter meant, as the cover advertised, “For co-editors and collaborators only” where he explained the methodology for loaning dried specimens, exchanging material, and general mutual cooperation “through a combined effort on an international basis.” (Van Steenis 1947, p. 4). At the end of 1948, the first pages of the Flora had been published, including an introduction to the project, as well as full enumerations of a few smaller families done by botanists close to Van Steenis (Van Steenis 1948).

Although Van Steenis had been working towards this passion project for years, it was not until he began cooperating with Indonesian scientific administrators in 1949 that he was able to secure funding for this international flora. While Wisaksono was in Europe in mid-1949, he met with Van Steenis, and they agreed to create a dedicated foundation which would run the project.^18^ In late 1949, just as Dutch power in Indonesia was coming to an end, Van Steenis went to Bogor, where he stayed through the middle of 1950, to iron out the details of this new effort. In 1950, Indonesian bureaucrats and politicians agreed to fund the Flora Malesiana Foundation, as long as an Indonesian scientist was its director, even with the understanding that Van Steenis would be its chief researcher.^19^ Koesnoto was appointed the director of the foundation later in 1950.^20^ Koesnoto acknowledged that as long as few Indonesian scientists were available to contribute directly to this effort, enlisting foreign botanists would be needed to begin the project.^21^ Nonetheless, the Flora Malesiana was an Indonesian scientific project which was internationally oriented and open to participation from scientists worldwide. It would make Indonesian science a showcase for international achievement through its sponsorship of a novel and important new flora. Van Steenis summarized the position of the Indonesian government: “They know that the whole world is dependent upon each other, and that precisely their independence enhances this dependence.”^22^

In Indonesia, the Flora Malesiana was a means for Indonesia’s entry into global science. In October of 1950 the final bylaws of the Flora Malesiana were approved by Koesnoto, Hermen Kartowisastro, then Director of Agriculture and Fisheries (later just the Department of Agriculture), and Djuanda Kartowisastro, Minister of Prosperity, who had ultimate authority over the Kebun Raya: “As the initiative, first plans, and first progress towards a ‘Flora Malesiana’ occurred in Java and as further activities in this field will be made possible by a Government grant to the Foundation, the Foundation shall be an Indonesian organization.”^23^ The Indonesian Department of Agriculture allocated 100,000 Dutch guilders per year for the Flora Malesiana, most of which went to pay salaries of Van Steenis and his collaborators in Holland, and to subsidize the publishing of the *Flora Malesiana*.^24^ When in 1950 the first substantial publication from the Flora Malesiana Foundation appeared, the frontispiece prominently included the seal of the Republic of Indonesia, and explained that it was:

Published under the auspices of the Kebun Raya Indonesia, Bogor, Java, Botanic Gardens of Indonesia, Bogor (Buitenzorg) and of the Rijksherbarium, Leyden, Netherlands. Prepared on an international co-operative basis under the supervision of several directors of botanic gardens, keepers of herbaria and various prominent botanists. For the promotion of botanical science and cultural advancement of the peoples of South-Eastern Asia to the Southwest Pacific Region. (Van Steenis-Kruseman 1950, p. iii)

In early 1951, Koesnoto wrote that internationally credible botanical research based on the Bogor herbarium collections was, after years of interruption due to war, revolution, and political uncertainty, again underway in Indonesia.^25^ With the successful launch of the *Flora Malesiana,* Koesnoto argued that he could now build a real international network of tropical botanists interested in Southeast Asian flora.^26^ The Flora Malesiana project, as a 1952 English language publication from the gardens made clear: “has been established … *on an international basis*, for the simple reason that plant distribution is utterly independent of political boundaries” (Bogor Scientific Centre 1952, p. 13). This publication went on to enumerate the fifty-two botanists (3 Australian, 7 English, 3 French, 2 German, 7 Indonesian, 1 Malayan, 17 Dutch, 1 Scottish, 1 Swedish, 3 Swiss, and 8 American) who had signed on as cooperators. George Sarton, in a 1951 *Isis* review of Van Steenis-Kruseman’s encyclopedia of Malay collectors, spoke of the project highly, under the title “Science and Peace” (Sarton 1951). Koesnoto wrote in early 1951 to Van Steenis: “It appears that the Kebun Raya Indonesia shall extend its wings much further than the Botanical Gardens has ever done before.”^27^ Koesnoto had further reason for optimism, as UNESCO had that year begun building a permanent science office in Indonesia. In the context of that news, Koesnoto wrote that Bogor and its botanical scientific institutes were coming to be seen internationally as a center of science.^28^

## UNESCO’s Science

Koesnoto was referring to UNESCO’s Field Science Co-operation Offices (hereafter referenced as Science Offices) which had begun in 1947 under Joseph Needham’s direction. For Needham, these offices were critical to expanding the opportunities for science outside of the West. Needham’s goal was to bring science to the global peripheries of Asia, Africa, the Middle East, and South America, an idealistic effort to strengthen science by sharing scientific knowledge and empowering scientists in what we would now call the Global South (Mougey 2018; Petitjean 2006c).^29^ Needham’s science cooperation offices were established in the late 1940s in Montevideo, Cairo, New Delhi, and Nanjing (Florkin 1956).^30^ The entire objective of fostering scientific collaboration across political boundaries while also building scientific capacity at the edge of the industrial world—Needham’s “periphery principle”—had no playbook. UNESCO’s scientific staff adapted existing European ideas about building scientific cooperation, developed in the 1920s and 1930s, which included organizing international scientific congresses, sponsoring educational missions and exchanges, as well as encouraging international research collaborations (Laqua 2011). An early initiative of the UNESCO science department was to provide travel funds to scholars from Asia, Africa, and South America so they could make their own scientific contacts and attend international scientific conferences.^31^

In 1950 UNESCO’s science mission was enlarged to include organizing and funding their own regional meetings.^32^ In Asia, the first meeting was in Bangkok in November of 1951.^33^ Official representatives from national scientific organizations were invited to attend, with travel funds provided by UNESCO. Slamet Imam Santoso, a psychiatrist, represented Indonesia, as the official delegate from the Ministry of Education.^34^ UNESCO’s new scientific leadership attended the Bangkok meeting, including Pierre Auger, a French physicist, who was Joseph Needham’s successor as the head of the natural sciences department. This meeting is an early window into UNESCO’s ideas about global science under Auger’s leadership. The meeting briefed the Asian scientific representatives on the services provided by UNESCO, including the principles for the exchange of lecturers and publications, and the proper method for teaching science in schools. More generally the meeting was to show Asian scientific leaders how UNESCO would facilitate disseminating science throughout Asia and the Pacific. This agenda, more paternalistic than Needham’s goal of helping scientists in the Global South, was derived from the British Council and its affiliated Association for the Advancement of Science’s recent efforts to expand science in the British Empire and Commonwealth. For Auger, dissemination of Western knowledge to the still-developing regions of Asia was the goal of UNESCO’s science department. But that did include providing travel funding for scientists from newly decolonized countries.

Although perceived by Auger and UNESCO staff in Paris as a means for outreach to the periphery, the Global South, the Science Offices were held in high regard in their host countries. The Science Offices were at that time the only long-term physical UNESCO bureaus outside of Paris. In fact, other UN-affiliated agencies did not have anything comparable. Thus these Science Offices, even if their mandate was limited to science, were prestigious and important to the host country, even beyond the scientific assistance they could offer. Within their host countries, government officials regarded them as general UNESCO offices, and they did end up serving as a general liaison to UNESCO.^35^ This both blunted the effectiveness of the offices, as it took time to serve all of these other cooperative requests, but also established, at least in the countries and cities which contained a science office, that science was central to UNESCO’s mission.

In 1950, following a formal Indonesian government request, UNESCO decided to place one of these offices in Indonesia (UNESCO 1951).^36^ A major reason for the Science Office being placed in Indonesia was that by then Indonesians’ global scientific ambitions were already apparent. Shortly after achieving sovereignty, Indonesians had begun to actively use their recently reorganized Organization for Scientific Research (OSR) to reorient science in Indonesia towards global cooperation. The OSR had originated in 1947 and 1948 as a final effort by Dutch government scientists to coordinate colonial science within the colony. In 1949 it began publishing a science journal, in Dutch. In 1950, after the transfer of Dutch sovereignty (but while there were still many Dutch scientists in Indonesia), the publication switched to English, with a new name, *O.S.R. News*. The masthead in 1950 announced that it was mailed free of charge to scientific institutes around the world who maintained professional contacts with Indonesia. The OSR actively cultivated contacts with foreign scientific organizations, and started sending scientific representatives to meetings and conferences abroad. In 1950 and early 1951, the OSR directors were still mostly Dutch, but by the end of 1951, the majority of leadership positions were held by Indonesian scientists, including chairman of the general secretariat, who after April 1951 was F. K. Wawo Roentoe. Koesnoto became a member of the Governing Board of the OSR in September of 1951 (Broersma 1952).

## UNESCO, Science, and Indonesia

In March of 1951 UNESCO opened its Science Office in the OSR building in downtown Jakarta, under the leadership of the Hungarian biologist A. Wolsky.^37^ Wolsky traveled around Java in 1951 exploring the scientific institutions of Java, and in 1952 Wolsky was in Bogor and attended the celebration of the 135th anniversary of the founding of the Kebun Raya.^38^ Although Wolsky was also the UNESCO science officer for other UN member states in Southeast and East Asia, 39 and he spent some of his time in those countries, the Science Office’s presence in Jakarta meant Indonesian scientists had particularly good access to UNESCO’s science resources. For example, Indonesian scientists could access UNESCO funds to travel to international conferences. In 1954 UNESCO sponsored the medical pathologist from the University of Indonesia Sutomo Tjokronegoro on a tour that took him to Europe, North and South America, and other parts of Asia.^40^ More importantly, the UNESCO office assisted with organizing symposia and providing travel grants to individual scientists, from Indonesia and elsewhere, which allowed them to participate directly in scientific congresses where Indonesian scientists could present research and network with other colleagues. This included the 8th Pacific Science Congress, held in November of 1953 in the Philippines, to which the UNESCO science office provided travel stipends, allowing four Indonesian scientists to attend.^41^ The Jakarta office further sponsored and paid for a Medicinal Herbs Symposium to be held in conjunction with the larger meeting. In anticipation of this meeting, Koesnoto received funds and assistance to visit Manila earlier in the year, where UNESCO arranged meetings and tours with scientists, including the Philippines’ leading botanist Eduardo Quisumbing, who was arranging the pharmaceutical meeting in an effort to launch a coordinated Southeast Asian effort to analyze medicinally useful plants.^42^ Although Koesnoto did not attend the subsequent Manila meeting (he did travel to India for a FAO meeting), J. Douglas represented the Kebun Raya, and F.K. Wawo Roentoe, the head of the Indonesian OSR, also attended.^43^ Van Steenis was in Manila, and gave a presentation about the plants cultivated at the Kebun Raya in Bogor (Van Steenis 1955). Indonesian biologists did not present papers at the conference, but this trip provided them credible participation in an international scientific meeting, and set them up for future appearances at international scientific symposia.

UNESCO’s involvement in tropical botany expanded the professional network for scientists working in the Global South, including for those from Indonesia. This included A. Dilmy, head of the Bogor herbarium after early 1955, and his mentor A. J. G. H. Kostermans, who as the official Indonesian representatives attended the 1956 UNESCO-sponsored international conference on tropical botany in Sri Lanka (then still Ceylon).^44^ This was paired with a meeting on “Methods of Study of Tropical Vegetation,” which was organized by the New Delhi and Jakarta Science Offices, and brought together scientists from across tropical Asia to discuss cooperative research efforts. Together these meetings would outline “problems of the humid tropical regions and proposing research programmes for the investigation of these problems,”^45^ and was the beginning of the Humid Tropics project, described below. Dilmy and Kostermans, traveling on UNESCO’s budget, subsequently published their paper in the conference proceedings as “Research on the Vegetation of Indonesia,” in which they stressed the challenges of botanical research in Indonesia, and the need for bringing additional botanists to Bogor (Dilmy and Kostermans 1958). This was perhaps the first time a scientific paper published by an Indonesian scientist—in this case Dilmy—was included in an international botanical publication.^46^ There were further plans to publish Kostermans’s world bibliography of the plant family Lauraceae, and to provide funding for A. H. G. Alston, one of Van Steenis’s Flora Malesiana researchers in Holland, to travel to the US for herbarium research in 1957.^47^

This effort to expand the scientific network of tropical botany was coordinated by the Science Offices, which had continued their focus on facilitating international scientific cooperation, even after their creators, Needham and Huxley, left UNESCO. As late as 1955 the Science Offices remained the largest budget item of the Department of Natural Sciences, most of which went to staff salaries. These offices continued to disseminate scientific information and knowledge to UNESCO’s member states, through lectures and radio presentations, and to encourage scientific exchange more generally.^48^ By the mid-1950s the department of natural sciences came under pressure to utilize these offices more effectively. In new guidance issued by UNESCO Director General Luther Evans, he stressed that the Science Offices were to devote resources to “concrete programme execution of [UNESCO’s] Department of Natural Sciences,” not to general liaison work, and to only assist with the organization of symposia from “responsible scientific bodies.”^49^ This is part of a larger shift of UN agencies, including UNESCO, towards providing technical assistance to member states, in an effort to support economic development (Finnemore 1993; Andersen 2016).^50^ For example, Indonesia used UN technical assistance to build a government macroeconomic planning board for the purpose of modernizing the economy (Webster 2011). UNESCO’s science department received additional funds specifically intended for technical assistance, which in general went to paying for scientific training and research in member states. In 1957 for Indonesia, UNESCO paid for university lecturers in chemistry, physics, and physiology, using technical assistance funds not usually part of the regular budget of the science department.^51^

Despite the new focus across all the UN agencies on technical assistance, the natural science department’s chief Auger found ways to organize scientific cooperation directly under his authority. Throughout the 1950s he re-envisioned the Science Offices as a means for expanding UNESCO’s own international collaborative projects (Sewell 1975). This began in 1952 with the Arid Zone Programme, with the goal of using science to help solve the problems of living in the arid regions of the world. Two years later, the natural sciences department began its counterpart, the Humid Tropics Programme research project, to address the challenges of living in tropics. A third project started in the mid-1950s focused on marine science. By 1955, these projects were the prime mission of the Science Offices, so much so that in late 1955, senior managers at UNESCO insisted that three of the Science Offices (including Jakarta) revise their work plans for 1956 with more support for one of these three research efforts.^52^ In 1957, these international scientific research programs had become the largest component of the regular budget of the Department of Natural Sciences, surpassing that of the Science Offices.^53^ Moreover, the Science Offices were required to use most of their non-personnel budgets to support either technical assistance or the Department of Natural Science’s authorized research programs.^54^ The Humid Tropics project was a separate initiative from the Science Offices. But the Jakarta Science Office took an important part in implementing its program, including assisting in organizing its first big event, the conference in Sri Lanka in 1956, discussed above.^55^

## UNESCO’s Humid Tropics

The Humid Tropics was seen by Indonesian scientists as a boon, as it linked Indonesian botanists to a global community of tropical botanists, and brought new attention and opportunities to scientists at the Kebun Raya. It brought visiting botanists to Bogor, to assist on collection trips, but also to serve as teachers and mentors to the young Indonesian botanists (Doty 1964). By the mid-1950s, UNESCO’s Jakarta office and the Kebun Raya were for a few years important hubs of research and coordination. For example, in 1958, the Kebun Raya hosted a Humid Tropics symposium, in collaboration with the newly established Indonesian Council of Sciences and the UNESCO office, which paid for the travel of foreign scientists.^56^ This international scientific congress was organized by Koesnoto and his staff at the Kebun Raya in Bogor. There were scientists from across Asia, as well as Great Britain, Australia, and the United States. At the opening address, Koesnoto extolled the importance of a scientific symposium about “the Reconstruction, the Stabilization and the further Progress of the countries in the Humid Tropics regions, particularly those which have but not long ago gained their Independence.”^57^ He opened the scientific proceedings by reading his paper about the “Flora Malesiana and the Ecological Studies of the Tropical Vegetation,” which surveyed the progress made in investigating the vegetation of tropical Southeast Asia.^58^ Sarwono Prawirohardjo, the president of the Indonesian Council of Sciences, spoke of the great task of restoring the biological balance between nature, climate, soil, and humanity, and that this task “must be done on the basis of international cooperation” and that Indonesia would expand its capacity for contributing to this international undertaking.^59^ This was the first international scientific meeting to take place in Bogor, and a crowning achievement for Koesnoto, who could point out that Indonesia and the Kebun Raya were at the center of a scientific effort to improve global living conditions in the tropics. Amongst its resolutions, the symposium participants recommended continuing to promote global research exchanges of material, students, and researchers. The scientific work was to focus on collecting new botanical material, and that across the Southeast Asian region, this material would be processed “according to an agreed-upon scheme of description and classification.”^60^

The 1958 symposium was an important boost for Indonesian botany, especially as at that time Indonesian scientists in Bogor were expanding educational opportunities for young Indonesian botanists. In the late 1950s the first graduates from a specialized biology college begun by Koesnoto began working at the Gardens, training to be the next generation of Indonesian botanists (Sastrapradja 1999). And the Jakarta Science Office supported these efforts. In 1961, they sponsored a botanical training expedition in the region around Bogor for Indonesian botany students, which then led to at least four subsequent UNESCO training trips in various parts of Southeast Asia. These collections trips became an important way for Indonesian and Southeast Asian botanists to train younger scientists on botanical practices in tropical Asia.^61^ Many of these students went off to graduate training in Europe and North America in the 1960.^62^

Concurrent to the opportunities provided to Indonesian scientists, the Humid Tropics project also helped to bring about the centralization of tropical botany in the West. The project was run by P. A. Varughese at the UNESCO headquarters in Paris. His work was overseen by an international advisory committee of scientists.^63^ Humid Tropics initially included participation and expertise from all over the world, represented by the diverse membership of its advisory committee. In 1963 the incoming director of the Bogor Kebun Raya, Otto Soemarwoto, was chosen as the Vice-Chairman of the advisory committee.^64^ But by then, the center of gravity of Humid Tropics had largely shifted towards tropical botanists in the West. The advisory committee was supposed to steer the scientific direction of the Humid Tropics program, but by the late 1950s, it did little more than validate UNESCO decisions made in Paris. And many of the committee’s unsolicited recommendations were ignored by Varughese and UNESCO.^65^ By the early 1960s, the main responsibility of the advisory committee became assigning research funds, which by 1961 went largely to Western botanists, facilitating their travel to tropical field sites, usually couched in terms of aiding their cooperation with scientists in the tropics.^66^ While the involvement of botanists from the Global South, including Soemarwoto, suggested global leadership, Humid Tropics provided a means for Western control over the global research agendas.

A watershed was in 1961, when the Humid Tropics project got a de facto steering committee of Western scientists. A year earlier the chair of the advisory committee, F. R. Fosberg, a specialist on the flora of Pacific region and then still at the US Geological Society, convinced Varughese to create a Visiting Committee for tropical herbaria.^67^ For Fosberg the new Visiting Committee was a way for Western botanists to manage the Humid Tropics effort to suit the needs of Western institutions. The Visiting Committee included Varughese and Fosberg, as well as A. C. Smith, director of the Museum of Natural History of the Smithsonian, G. Taylor, Director of the Royal Botanic Garden at Kew, and H. J. Lam, the director of the Rijksherbarium in Leiden.^68^ Although innocuous sounding, Fosberg’s proposal to Varughese makes clear that this committee’s charge would be far-ranging, by providing guidance, support, and recommendations to tropical herbaria, to facilitate exchanges of specimens and other material, and to “represent the interests of the international botanical public in these institutions.” The context makes it clear that the use of the word “international” meant Western. Their first project was preparing and distributing a manual for herbarium practice, but it was to include issuing specific recommendations to the governments housing tropical collections about means of improving and maintaining the collections, so it would facilitate use by visiting “international” botanists.^69^ The first meeting was held at the Rijksherbarium in Leiden in May of 1961. In addition to the Varughese and the four committee members, Van Steenis was there on behalf of the Flora Malesiana, as well as Frans Stafleu from Utrecht University, representing the administrative bureau of the International Association of Plant Taxonomists. The group decided on a series of functions for the Visiting Committee, which was to advise local authorities on best practices, to facilitate easier shipment of specimens from tropical herbaria, and to provide technical consulting to herbaria as needed.^70^ Although left unsaid, this was a committee of American and European scientists to direct and manage an international effort in plant taxonomy.

The Visiting Committee also sought to create an international effort to write the “Flora Neotropica,” a publication series about the flora of the tropical Americas, along the model of the Flora Malesiana. After the committee members visited a number of South American herbaria on the way to Brazil, the first visiting committee meeting in São Paulo was devoted to planning this new flora.^71^ There was apparently some grumbling from South Americans about how the Flora Neotropica would be managed, but the only concern raised internally by UNESCO about the diversity of the Visiting Committee members was its lack of a French botanist.^72^ This meeting established the pattern of the Visiting Committee. It provided written evaluation of herbaria in the tropics, made recommendations to UNESCO and the community of botanists on their strengths and shortcomings, and sought to strengthen the ties between European, North American and tropical institutions.^73^ The 1963 meeting in Singapore continued this theme, where the Western botanists were using UNESCO funds to try to organize an international network, ultimately centered on the herbaria in Leiden, London, and Washington.^74^ At that time, Indonesian scientists were still looking to Humid Tropics and UNESCO as a way for advancing Indonesian science, and Sarwono, who had become the head of a federal agency that now included all national scientific institutes, including the Kebun Raya, was at that time disappointed to learn that the meeting was not going to be in Indonesia.^75^ Just prior to UNESCO ending the Humid Tropics program (and thus the visiting committee), UNESCO launched the Organization of Flora Neotropica, headquartered at the New York Botanical Garden (Fosberg 1985). The other result of the visiting committee was the publication of the *Manual for Tropical Herbaria* in 1965, paid for by UNESCO and published under the auspices of the International Bureau for Plant Taxonomy and Nomenclature, from its office at Utrecht University (Fosberg and Sachet 1965). The Humid Tropics effort ended in 1964, but by then it had already facilitated the efforts of scientists such as Fosberg, Stafleu, and Smith, to adapt the networks of UNESCO’s Humid Tropics to allow them to gain authority over global tropical botany, and utterly dismiss the fostering, let alone funding, of strong scientific networks across tropical nations.

UNESCO continued to sponsor science after 1964, although at a smaller scale, and largely organized through technical assistance grants, intended to promote economic development. Soemarwoto, after 1964 director of the Kebun Raya Indonesia (which was then officially known as the Lembaga Biologi Nasional), continued to build international scientific cooperation centered on Bogor through contacts with Southeast Asia, Europe, and the United States. He was able to secure a West German grant in the mid-1960s, which he used to supplement the operating budget at a time of diminished government funding.^76^ And in 1968, Soemarwoto created the Southeast Asian Regional Centre for Tropical Biology, which was funded by the Association of Southeast Asian Nations and the United States, and was an effort to continue the global research agendas of the 1950s in the context of Southeast Asia. It lasted for three years as an independent entity, before being taken over by the Indonesian government (Soemarwoto 1970a, b). Subsequently the Indonesian scientists in Bogor worked towards establishing a national research institute in service to the Indonesian national needs, especially as it related to agricultural development (Goss 2011).

## Global Science

The ideal of open international scientific collaboration across all UN-member nations began to unravel at the end of the 1950s, under pressure of the Cold War, but also as Western scientists asserted control over their disciplines, from their academic perches in Europe and North America. And at the same time, scientists in newly independent nations in the Global South noticed that they were once more being engaged as servants rather than as respected partners. An early indicator of Indonesia’s withdrawal from international science was in 1958, when Indonesia stopped paying for the Flora Malesiana. Prior to that it had been fully funded by the Indonesian government. In late 1957, as a result of increased political tensions between Indonesia and the Netherlands, including over continued Dutch control of West Irian, Indonesia severed diplomatic relations with the Netherlands. This made it virtually impossible for Koesnoto to pay Van Steenis, although in 1958 Koesnoto managed to partially fund the Flora Malesiana via an intermediary in Brussels.^77^ By then it seemed clear to the Indonesian government that the Flora Malesiana was not, and perhaps never had been, in the hands of Indonesian scientists. So when the government cast a jaundiced eye on Dutch activities, the Flora Malesiana was understood to in fact be under the control of Dutch scientists. While that was the end of Indonesian government budgetary support for the Flora Malesiana, the project continued to be a focus of collecting, processing, and research activity at the Kebun Raya through the early 1960s, and botanists in Leiden and Bogor were able to exchange herbarium material via the Copenhagen herbarium.^78^ Nonetheless, the Flora Malesiana now was controlled solely by Van Steenis. Between 1959 and 1962, he received some temporary funding from the Dutch Research Council and the Rijksherbarium. After Lam’s retirement as Rijksherbarium director in 1962, Van Steenis succeeded him. With that, the Flora Malesiana became an official activity of the Rijksherbarium. Under Van Steenis’s direction, the Rijksherbarium became a global center of tropical biology, a hub that included herbaria in the West as well as the Global South.

Despite decolonization, Western botanists, some of them veterans of imperial science, established a large measure of authority over global tropical botany in the 1960s. This study of that history in Indonesia shows continuity from pre-1945, as global botany was brought under Western authority. Nonetheless, the architects of this system, Western scientists such as Auger, Fosberg, and Van Steenis, did not recreate imperial science and its practices. Instead, they leveraged the language, prestige, and institutions of internationalism to center scientific authority in the West. This was an imperialized and even exploitative form of authority, where scientists in the Global South were patronized by scientists in the West. Furthermore Western scientific institutions exerted control over publishing and disseminating of scientific knowledge, even when the data came from the tropics such as with the Flora Malesiana. Institutions in the Global South like the Kebun Raya, while important as sites of collecting and processing of data and collections, were subservient to scientific centers of publishing, validation, and global authority in the West.

By the late 1950s, efforts to build international centers of science outside of Europe had begun to stall more generally. Some of this was due to a broader shift from international cooperation to national scientific development in decolonizing countries (De Greiff 2002). But it was also a direct result of the UNESCO scientific leadership’s change in vision for global science. In the mid-1950s, UNESCO’s science policy shifted from supporting scientific research and collaboration in the Global South to running their own global research projects. Under the leadership of Pierre Auger, who directed the natural sciences department from 1948 until 1959, UNESCO moved towards erecting what he subsequently identified as the most important element of modern science, an administrative machinery for coordinating global science (Auger 1962). Under Auger, model research institutions were places like the European Organization for Nuclear Research (CERN), a complex international scientific institute in Geneva, which Auger had played a key role in creating in the early 1950s (Auger 1963; Petitjean 2006b). UNESCO helped position European and North American institutions at the center of global science, now with a paternalistic relationship to science and scientists in the Global South. For tropical botany, this meant that UNESCO was deliberately supporting efforts by Western botanical institutes to control the direction of the field, through efforts such as the Visiting Committee, which was made up exclusively of botanists based in Europe and North America.

As a result of UNESCO’s changing scientific policies, Indonesian tropical biologists were treated as peripheral and secondary, eligible to attend meetings and receive training funds, but not able to set the scientific research agendas. At the same time, Indonesia’s internationalization initiatives began to change. By the late 1950s, Indonesia’s efforts to chart an independent political course increasingly set it at odds with the US and its European allies, and drew it closer to China and the Soviet Union. Sukarno’s first visit to China in 1956 made a big impression on him, and gave him a model and justification for replacing a parliamentary system with Guided Democracy while also steering Indonesia away from the West (Liu 1997). Many Dutch citizens had continued to live and work in Indonesia after independence, but this ended in 1957, when Dutch businesses were nationalized, and Dutch citizens, including the remaining scientists, were expelled from the country. Tensions with the Netherlands continued to mount, almost leading to a war over the West Irian territory in 1962. After that, Sukarno’s foreign policy was openly hostile to the West, and he subsequently sought to block the merger of the Federation of Malaya with formerly British colonies in Borneo. This led to armed conflict, now between Indonesian and British forces, and Indonesia began receiving military aid from China and the Soviet Union (Jones 2002). Sukarno in early 1965 withdrew Indonesia from the United Nations, arguing that Malaysia receiving a Security Council seat was evidence of the UN’s support of neo-colonialism. Indonesia remains the only country to ever withdraw from the UN. Sukarno’s international policy under Guided Democracy imperiled Indonesia’s collaboration with the West, ultimately eroding the ability of Indonesian scientists such as Soemarwoto or Sarwono to cooperate with European or North American scientists.^79^

During the 1960s, two tiers of scientific authority developed within tropical botany, one within the nations of the Global South, and another with a world-wide mandate and reach. Global science brought collections, researchers, and funding from across the world to a few central institutions in the West. Tropical botany was led not just from the old imperial centers such as Kew Gardens, but at new sites of global science such as Leiden University, the Smithsonian Institution, and the New York Botanical Garden, all of which dramatically expanded their collections and scientific research on tropical botany in the 1950s and 1960s. At the same time, Cold War politics created barriers, alliances, and hierarchies that made the idealistic scientific collaboration envisioned in the early 1950s impossible. In Indonesia this led by the late 1950s to a slow retreat from international scientific cooperation. After 1966, however, the new political regime of Suharto rekindled ties with the West, which led to a return of scientific exchange. Scientific research in Bogor expanded rapidly after 1970. The young leaders of the Kebun Raya, all with graduate training in the West, positioned the Kebun Raya as a national research center, and as such integral to the development of the Indonesian nation (Rifai et al. 1975). Under the directorship of Setijati Sastrapradja in the 1970s and 1980s, the budget of the Bogor National Biological Institute increased twenty-fold in a little over ten years, largely a result of the Bogor institute’s role in utilizing Indonesia’s plant resources more productively, to serve national interests (Goss 2011). Throughout the 1970s and 1980s, there was still a vital link to global science, with numerous Western scientific visitors in Bogor, and renewed participation in the Flora Malesiana by Indonesian researchers. And it continued to be possible to jump from Indonesian botany to global botany—especially through graduate training in the West. Nonetheless, these were different scientific cultures, and by the 1990s, they had drifted apart. Although beyond the scope of this paper, a more recent development has been closer cooperation between Indonesian botanists and botanists from elsewhere in Southeast Asia.

## Conclusion

Decolonization broke established imperial circuits of knowledge and expertise. And that meant newly sovereign nations had new opportunities to establish their legitimacy within science, especially in fields such as tropical botany where they now administered established research institutes. In Indonesia in the early 1950s, Indonesian scientists found wider collaboration with colleagues outside Indonesia, now with them overseeing the herbarium collections; and, able to collect new material, they saw a bright future for Indonesian science. This ethos of global scientific cooperation found ready support in UNESCO, where an explicit goal of Needham and Huxley had been building an international world of science that empowered scientists from formerly colonized nations. Indonesian scientists moved quickly to establish Indonesia as a global center for tropical botany. Indonesian scientists and government officials funded an innovative international research project—the Flora Malesiana—and attracted a UNESCO science office to Jakarta. Indonesian scientists began attending international conferences, and began to publicize their scholarship to a global audience. UNESCO was initially a willing and enthusiastic partner. The UNESCO science officials in Jakarta and Paris facilitated Indonesian scientists entering into global collaborations, especially by providing travel funding. And the Humid Tropics program, focused on tropical flora, was seen by Indonesian scientific leaders as a great way to showcase their contributions to tropical botany, and provided both national and international legitimacy for their efforts.

The emerging model of global science after 1960—in tropical botany but in other disciplines as well—was the Western-based and funded research center with a global mandate, with its own funding, students, collections, laboratories, and publications. As the history of Indonesian botany during the 1950s analyzed in this article makes clear, decolonization and UNESCO’s early internationalism did not lead to new centers of global science taking root in the Global South. Instead decolonization integrated scientists from the Global South, as well as their collections, students, research projects, and institutions, into larger imperialized networks of scholarship. This model of research, which Auger and UNESCO implemented in the 1950s and was largely in place by 1960, was theoretically for everyone—and in fact did employ non-Western scientists—but was controlled and managed in the West. And it saw numerous scientists from the Global South leave their home countries to pursue science in the West, contributing to the “brain drain” that by the 1960s was of serious concern to officials in Asia, Africa, and Latin America, and was catalogued in a UNESCO preliminary report in 1968.^80^ By the 1960s, at a time of increased global scientific mobility, exchange, and collaboration, and when independent African countries began their journey to global science, the mold was set. European and North American institutions managed and directed global science, even in disciplines like tropical botany, where the collections and applications were mostly in the Global South.

## Footnotes

1 It was also apparent in the global engagement by Indonesian artists and intellectuals in the 1950s, who found ways to create a new national Indonesian culture in a global context (Lindsay and Liem 2012).

2 Although Bogor was a hotbed of revolutionary activity in the first year of the revolution, it came under full Dutch control by late 1946.

3 Wisaksono Wirjodihardjo to Hoofd van de Dienst voor Algemeen Personele Zaken, December 11, 1948, file 869, in Algemeene Secretarie 1944–1950, Arsip Nasional Republik Indonesia, Jakarta (hereafter, AS-ANRI).

4 Van Steenis to Schuurman, August 6, 1949, file 8, Van Steenis papers, Nationaal Archief, The Hague (hereafter, Van Steenis papers).

5 Van Slooten and others had since late 1945 sought to secure even greater control by the Dutch scientists at the Botanical Gardens over Indonesian science (Van Slooten 1949).

6 It continues to this day to describe itself as an international research project: https://floramalesiana.org.

7 Van Steenis had always envisioned that this flora would describe and catalog the plants of tropical Asia, including those in British Malaya and the American Philippines. In the 1930s he had written a number of exploratory essays about the plant-geography of Southeast Asia, which laid out the intellectual motivation for such a flora (Van Steenis 1934, 1935a, 1936; Jacobs 1972).

8 Verdoorn and Van Steenis were friends from university and were in regular correspondence.

9 Lam to Van Steenis, February 12, 1935, Buitenzorg correspondence archive 1933–1945, Leiden Herbarium library collections, Naturalis Biodiversity Center.

10 Van Steenis to Van Slooten, October 16, 1946, file 8, Van Steenis papers.

11 Van Mook, “Memorie van Inlichting,” Volksraad, zittingsjaar 1939-1940, “Zevende aanvullende begrooting betreffende afdeeling VI der begrooting van Nederlandsch-Indie voor 1940,” Onderwerp 126, February 1940, Ministerie van Koloniën, Geheim Archief 1901-1940, Geheim Verbaal, March 30, 1940/Y19, no. 580

12 C. G. G. C. van Steenis, “Blown by the Wind, learnings through the years. Autobiographical notes,” March 1986, file 30, Van Steenis papers.

13 Van Steenis to Verdoorn, November 5, 1945; also Van Steenis to Verdoorn, September 12, 1945; both in Van Steenis file, Descartes Centre for the History and Philosophy of the Science and the Humanities, Utrecht University.

14 Bloembergen to Van Steenis, February 9, 1949, file 5, Van Steenis papers. There was no funding for the Flora Malesiana in the Dutch colonial budget of 1947 or 1948. Honig to Van Steenis, July 31, 1948, file 6, Van Steenis papers.

15 D. F. van Slooten, then director of the Botanical Gardens, wrote Van Steenis in the middle of 1948 that he was opposed to the Flora Malesiana project. Van Slooten to Van Steenis, June 8, 1948, file 8, Van Steenis papers.

16 A UN Security Council resolution in January 1949 called for a speedy Dutch retreat, and the creation of an interim government including Republican leaders. At the same time, the United States told the Dutch it would withhold Marshall Aid if they did not abide by the UN resolution. This threatened not just the economic recovery of the Netherlands, but their strategic alliance with the United States, including participation in NATO. By late April, the Dutch had agreed to a ceasefire, and the Republic’s participation in a federal Indonesia. Sukarno returned victoriously to Jogjakarta on July 6, 1949 (Reid 1974).

17 Van Steenis, “Gespecifeerde uitgaven in Nederland verbonden aan het werk Flora Malesiana,” 1948, file 269, AS-ANRI, and Van Steenis, “De langdurige barensweeën van Flora Malesiana 1690-hodie,” 1947, file 197, Van Steenis papers.

18 Van Steenis to Schuurman, September 21, 1949, file 8, Van Steenis papers, and Merrill to Van Steenis, September 14, 1949, file 161, Van Steenis papers.

19 Van Steenis to De Wit, March 16, 1950, file 9, Van Steenis papers.

20 Van Steenis to De Wit, August 27, 1950, file 9, Van Steenis papers.

21 Koesnoto, “Kebun Raya Indonesia Bogor, Rentjana Pekerdjaan 1950,” file 178, Van Steenis papers.

22 Van Steenis to De Wit and Lam, July 4, 1950, file 9, Van Steenis papers. In a separate effort to placate concerns from Dutch scientists, who were deeply skeptical of Indonesians’ ability to lead a major scientific project, Van Steenis argued that because he was the editor of the Flora Malesiana series, and the name was owned by the publisher Noordhoff-Kolff, Indonesians would *not* have final scientific say over the content of the work. There is no indication Van Steenis made this clear to Koesnoto or others in Indonesia.

23 “Explanatory memorandum to the Deed establishing the Foundation ‘Flora Malesiana,’” October 21, 1950, file 160, Van Steenis papers.

24 See for example, C. G. G. J. van Steenis, “Tenth Semi-Annual Report of Flora Malesiana Foundation, July-December 1955,” January 1956, file 61, Van Steenis papers, listing a budget for 1955 of f. 89,695.64.

25 Van Steenis to Koesnoto, June 6, 1951, file 178, Van Steenis papers.

26 Koesnoto, “Kebun Raya Indonesia Bogor, Rentjana Pekerdjaan 1950,” file 178, Van Steenis papers.

27 Koesnoto to Van Steenis, January 30, 1951, file 178, Van Steenis papers.

28 Koesnoto, “De toekomst van de Regeringsinstellingen voor natuurwetenschappelijk onderzoek to Bogor,” July 13, 1951, file 246, Van Steenis papers.

29 I will use the term Global South, those parts of the world with underdeveloped industry and technology, even though that terminology was not used in the early post-war period I am examining.

30 “Field Science Co-Operation Offices,” June 12, 1947, Natural Science/28, UNESCO archives.

31 By the mid-1950s, the Science Offices mission was formalized to encompass the following: facilitating general science liaison work, developing contacts between scientists in different countries, analyzing difficulties encountered by scientists, exchanging information, and building up directories of scientists and scientific institutions. B. Cacciapuoti to Malcolm Adiseshish, Function of the Science Co-operation Offices, June 2, 1955, 18.748, FSCO-General, Part III (1953–1954) file, UNESCO Archives (hereafter cited as Cacciapuoti, Function of the Science Co-operation Offices).

32 This was decided at the fifth session of the General Conference of UNESCO in 1950 and at a follow-up meeting of the Association for the Advancement of Science in Paris later in the year. Bodet to UK Commission for UNESCO, October 17, 1951, 231.831, Regional Meeting of AAS in Asia file, Nov. 1951, UNESCO Archives.

33 This meeting was planned in Paris by the natural science department, with Børge Michelson serving as the regional coordinator. R. Skénasi to B. Michelsen, November 14, 1951, NS Memo 9028, Regional Meeting of AAS in Asia file, Nov. 1951, UNESCO Archives.

34 “Summary Report of the First Regional Meeting of Associations for the Advancement of Science, Bangkok, November 28th–December 3rd,” December 15, 1951, Regional Meeting of AAS in Asia file, Nov. 1951, UNESCO Archives.

35 W. J. Ellis, “Functions of Science Co-operation Offices,” 1955, FSCO-General, Part III (1953–1954) file, UNESCO Archives.

36 In 1951, the Chinese office, which had formerly been in Nanjing, and then temporarily in Shanghai and Manila as a result of the Chinese revolution, was formally moved to Jakarta.

37 In 1952 the UNESCO office moved to its permanent location at Jalan Diponegoro 27 in Menteng, Jakarta.

38 “Rapport aux Nations Unies pour 1951: La régionaux de Coopération scientifique,” FSCO-General, Part II (1950–1952) file, UNESCO Archives, and “Activités au course de l’année 1951 et du premier trimester 1952 et projets d’activités envisages,” July 1952, FSCO-General, Part II (1950–1952) file, UNESCO Archives.

39 The Jakarta Office served Thailand, the Philippines, Laos, Cambodia, Vietnam, Japan, Korea, and East Pakistan, “UNESCO Regional Science Co-operation Offices,” February 6, 1952, FSCO-General Part II (1950–1952), UNESCO Archives. The Cairo office serviced West Pakistan. The Delhi office was responsible for India, Burma, and Ceylon (Sri Lanka). Wolsky resigned from UNESCO in 1954 to take a job at Fordham University, and he was replaced by J. Smid, who had previously led the UNESCO science mission at the Manila office, which was under the Jakarta office.

40 “Science Co-operation Offices. Symposia, Congresses, and Regional Training Courses,” 1954, FSCO-General, Part III (1953–1954) file, UNESCO Archives.

41 J. Smit to R. Adams, October 20, 1953, 1580, Medicinal Herbs Symposium 1953 file, UNESCO Archives.

42 “Extract of letter from Virginia Rice concerning Dr. Koesnoto’s visit,” 1953, Medicinal Herbs Symposium 1953 file, UNESCO Archives.

43 R. Sekanzi to R. Adams, January 8, 1954, 15.0126, Medicinal Herbs Symposium 1953 file, UNESCO Archives.

44 W. Ellis to J. Lebrun, November 20, 1955, 2686/SF, Humid Tropics—Herbaria and Zoological Collections, Part I up to 1960 file, UNESCO Archives; P. Auger to M. Jean Thomas, February 28, 1956, 22473, Programme & Budget—Department of Natural Sciences, Part II (1950–1960) file, UNESCO Archives.

45 “Activities Report of the Humid Tropics Programme for the period 1 January to 29 February 1956,” Programme & Budget—Department of Natural Sciences, Part II (1950–1960) file, UNESCO Archives.

46 Kostermans was at that time still a Dutch citizen. He became an Indonesian citizen in 1959.

47 “Work Plan for 1957 and 1958: Project 2315 Humid Tropics Programme,” February 15, 1957, Programme & Budget—Department of Natural Sciences, Part II (1950–1960) file, UNESCO Archives.

48 “Division de la cooperation scientifique,” 1951, FSCO-General Part II (1950–1952) file, UNESCO Archives.

49 Luther Evans, “Second Semester Work Plan Natural Sciences,” June 21, 1955, file Programme & Budget—Department of Natural Sciences, Part II (1950–1960), UNESCO Archives.

50 “Summary Record of Meeting Held on Monday, 1 March 1955,” March 9, 1955, 14.004, FSCO-General, Part III (1953–1954) file, UNESCO Archives.

51 “Chapter 2—Natural Sciences, 1959–1960,” February 6, 1959, Natural Sciences-Programme & Budget & Organisations, Part II (1950–1960) file, UNESCO Archives.

52 “Work Plan Review: Field Science Co-operation Offices,” December 2, 1955, FSCO-General, Part III (1953–1954) file, UNESCO Archives.

53 “Natural Science’s Budget during the Past Ten Years,” April 29, 1958, NS 75, Programme & Budget—Department of Natural Sciences, Part II (1950–1960) file, UNESCO Archives.

54 Lennart Mattsson, “Outline of the 1959–1960 Proposed Programme,” Jakarta, July 24, 1957, Programme & Budget—Department of Natural Sciences, Part II (1950–1960) file, UNESCO Archives. Mattsson had replaced Smid in mid-1957 as head of the Jakarta office.

55 Cacciapuoti, Function of the Science Co-operation Offices.

56 L. Mattsson to A. Establier, October 22, 1958, 577; Evans to Sen, November 20, 1958, 811.867; L. Mattsson to V. Veronese, December 31, 1958, Symposium on Tropical Vegetation—1958—Bogor—Indonesia file, UNESCO Archives. UNESCO Director General L. Evans wrote to the FAO director general B.R. Sen to encourage FAO participation.

57 L. Mattsson, “UNESCO Science Cooperation Office for Southeast Asia, Report: Symposium on the Vegetation of the Humid Tropics, December 12–15, 1958,” Symposium on Tropical Vegetation—1958—Bogor—Indonesia file, UNESCO Archives.

58 L. Mattsson, “UNESCO Science Cooperation Office for Southeast Asia, Report: Symposium on the Vegetation of the Humid Tropics, December 12–15, 1958,” in Symposium on Tropical Vegetation—1958—Bogor—Indonesia file, UNESCO Archives.

59 Sarwono Prawirohardjo, “Address of the President of the Council for Sciences of Indonesia,” December 1958, Symposium on Tropical Vegetation—1958—Bogor—Indonesia file, UNESCO Archives. Sarwono, an obstetrician, was a long time nationalist and during the revolution had started the University of Indonesia in 1945, and started the Indonesian Council of Sciences in 1956 (Pols 2018).

60 F. R. Fosberg, “Symposium on the Vegetation of Humid Tropics: Resolutions,” Symposium on Tropical Vegetation—1958—Bogor—Indonesia file, UNESCO Archives.

61 These subsequent trips were also sponsored by the Jakarta Science Office, and included participants from all over Southeast Asia, with only one or two Indonesian students participating. L. Mattsson to V. Kovda, April 16, 1962, 373.62, and Hsuan Keng, “A Report to the Director, UNESCO Science Cooperation Office, on the UNESCO Limestone Expedition to Ule Kelantan,” August 1962, Humid Tropics—Herbaria and Zoological Collections, Part II 1961 to 1962 file, UNESCO Archives.

62 By 1960 Soegeng Reksodihardjo, who was a member of the first cohort to graduate from Koesnoto’s biological college, was at Harvard pursuing graduate studies in botany. Kostermans to Van Steenis, July 25, 1960, file 76, Van Steenis papers.

63 “Chapter 2—Natural Sciences,” 1959/60 budget, Programme & Budget—Department of Natural Sciences, Part II (1950–1960) file, UNESCO Archives.

64 “Humid Tropics Research Programme, Advisory Committee for Humid Tropics Research, Fourth Session, Bandung (Indonesia), 2–6 December 1963,” NS/186, UNESCO Archives.

65 F. R. Fosberg to M. Batisse, April 27, 1964, Humid Tropics—Herbaria and Zoological Collections, Part III 1962 to 1964 file, UNESCO Archives.

66 “Advisory Committee for Humid Tropics Research, 3rd session, Honolulu, USA, 1961,” NS/HT/94 and “Humid Tropics Research Programme, Activities Report for the period November 1959 to July 1961,” NS/172, UNESCO Archives.

67 Fosberg moved to the Smithsonian Institution in 1966.

68 V. Kovda to G. Taylor, June 29, 1960, NS52/157, Humid Tropics—Herbaria and Zoological Collections, Part I up to 1960 file, UNESCO Archives.

69 F. R. Fosberg to P. A. Varughese, June 21, 1960, Humid Tropics—Herbaria and Zoological Collections, Part I up to 1960 file, UNESCO Archives. Fosberg had since the 1956 Ceylon conference been involved with Humid Tropics.

70 P. A. Varughese to V. Korda, May 23, 1961, 10, Humid Tropics—Herbaria and Zoological Collections, Part II 1961 to 1962 file, UNESCO Archives.

71 See for example, A. C. Smith, “Reports on Visits to Herbaria to Lima, Peru,” October 1962 and F.R. Fosberg, “Herbarium of the University of Costa Rica,” October 1962, Humid Tropics—Herbaria and Zoological Collections, Part II 1961 to 1962 file, UNESCO Archives.

72 Fosberg seems to have been successful at preventing the appointment of a French botanist to the committee, using the argument that the French herbaria were antiquated and that the most qualified French botanist, Alicia Lourteig, talked too fast and did not listen (making also clear she was a woman). F. R. Fosberg to M. Batisse, August 25, 1964, Humid Tropics—Herbaria and Zoological Collections, Part III, 1962 to 1964 file, UNESCO Archives.

73 F. R. Fosberg, “Report of the Second Meeting of the UNESCO Visiting Committee for Tropical Herbaria,” January 20, 1963, Humid Tropics—Herbaria and Zoological Collections, Part III 1962 to 1964 file, UNESCO Archives

74 F. R. Fosberg, “Report of the Third Annual Meeting of the UNESCO Visiting Committee for Tropical Herbaria,” November 30, 1963, NS/HT 115A, Humid Tropics—Herbaria and Zoological Collections, Part III 1962 to 1964 file, UNESCO Archives.

75 L. Mattsson to F. R. Fosberg, April 30, 1963, 312.63, Humid Tropics—Herbaria and Zoological Collections, Part III, 1962 to 1964 file, UNESCO Archives.

76 Otto Soemarwoto, personal communication, June 14, 2001.

77 C. G. G. J. van Steenis, “Fifteenth Annual Report of Flora Malesiana Foundation,” 1959, file 61, Van Steenis papers.

78 Van Steenis to Minister van Buitenlandse Zaken, July 3, 1963, 1165, file 92, Van Steenis papers.

79 After 1966 Indonesia’s New Order government, under President Suharto, established a new direction in foreign affairs, and saw Indonesia not only return as a member state of the UN, but also led to closer ties between Indonesia and the West.

80 “The Problem of Emigration of Scientists and Technologists (‘Brain Drain’ or ‘Exode des Competences’),” February 29, 1968, SC/WS/57, UNESCO Archives.
